# Intensive Multiprofessional Rehabilitation Is Superior to Standard Orthogeriatric Care in Patients with Proximal Femur Fractures—A Matched Pair Study of 9580 Patients from the Registry for Geriatric Trauma (ATR-DGU)

**DOI:** 10.3390/jcm13216343

**Published:** 2024-10-23

**Authors:** Ulf Bökeler, Ulrich Liener, Hannah Schmidt, Nils Vogeley, Vanessa Ketter, Steffen Ruchholtz, Bastian Pass

**Affiliations:** 1 Department for Orthopaedics and Trauma Surgery, Marienhospital Stuttgart, Böheimstrasse 37, 70199 Stuttgart, Germany; ulrich.liener@vinzenz.de (U.L.); nilsfrederik.vogeley@vinzenz.de (N.V.); 2 AUC-Academy for Trauma Surgery, 80538 Munich, Germany; hannah.schmidt@auc-online.de; 3 Center for Orthopaedics and Trauma Surgery, University Hospital Giessen and Marburg, 35043 Marburg, Germany; ketter@med.uni-marburg.de (V.K.); ruchholtz@med.uni-marburg.de (S.R.); 4 Department of Orthopedic and Emergency Surgery, Alfred Krupp Hospital, Hellweg 100, 45276 Essen, Germany; bastian.pass@krupp-krankenhaus.de

**Keywords:** hip fracture, multiprofessional rehabilitation, orthogeriatric care, fragility fracture, registry

## Abstract

**Background:** Orthogeriatric treatment, which involves a collaborative approach between orthopedic surgeons and geriatricians, is generally considered to be superior to standard care following hip fractures. The aim of this study was to investigate additional effects of a geriatrician-led multidisciplinary rehabilitation program. **Methods:** In this matched paired observational cohort study, patients aged 70 years and older with a proximal femur fracture requiring surgery were included. Between 1 January 2016 and 31 December 2022 data were recorded from hospital admission to 120-day follow-up in the Registry for Geriatric Trauma (ATR-DGU), a registry of older adults with hip fractures. Out of 60,254 patients, 9580 patients met the inclusion criteria, 4669 patients received early multiprofessional rehabilitation (EMR) and 4911 patients were treated by standard orthogeriatric co-management (OGC). **Results:** Compared to standard orthogeriatric treatment, multiprofessional therapy significantly lowered the 7-day mortality rate (2.89% vs. 5.11%) and had a significant impact on walking ability seven days after surgery (86.44% vs. 77.78%). **Conclusions:** In summary, a geriatrician-led multiprofessional rehabilitation program resulted in lower mortality and improved walking ability than standard orthogeriatric care.

## 1. Introduction

The global number of hip fractures is projected to increase to 6.25 million by 2050 [1]. Patients over the age of 70 are particularly often affected [2] and hip fractures are associated with decreased quality of life, disability, increased mortality, and high disease burden [3,4,5,6]. In addition to the ramifications on the individual patient’s quality of life, there is a significant impact on the health and social system [7,8]. To address the growing medical and economic challenges, multiprofessional care pathways and orthogeriatric units were established in recent decades [9,10]. The underlying idea of multidisciplinary and multiprofessional rehabilitation of proximal femur patients is that the complexity of a frail patient with severe comorbidities often exceeds the skills of orthopedic surgeons. Several studies have shown the superiority of orthogeriatric co-management compared to standard care in terms of lower mortality [11,12,13] and better physical function after hip fracture [14,15]. The extent of these orthogeriatric programs varies from a twice-a-week consultation by a geriatrician on an orthopedic ward to an integrated orthogeriatric multiprofessional program with daily interprofessional treatment by geriatricians, orthopedic surgeons, specialized nurses, physiotherapists, occupational therapists, speech therapists, and psychologists. The aim of this study was to investigate the additional effect of geriatrician-led multidisciplinary rehabilitation on outcome parameters (mortality, postoperative complications, walking ability, and patient-reported quality of life) compared to standard orthogeriatric care.

## 2. Materials and Methods

The source of the data in the present analysis is the Registry for Geriatric Trauma (AltersTraumaRegister DGU^®^; ATR-DGU). The ATR-DGU is a large prospective, multicenter, standardized, and anonymous registry. The ATR-DGU was founded by the German Trauma Society (DGU) in 2016 and provides information on geriatric trauma patients aged 70 years and older with hip, periprosthetic, and peri-implant femoral fractures requiring surgery. The scientific oversight is carried out by the Working Committee on Geriatric Trauma Registry (AK ATR) of the DGU. The infrastructure for documentation, data management, and data analysis is provided by the Academy for Trauma Surgery (AUC—Akademie der Unfallchirurgie GmbH). Participating centers transmit pseudonymized patient data via a web-based application into a central database. Currently, certified hospitals from Germany, Switzerland, and Austria contribute to the ATR-DGU, with a total of nearly 60,000 cases from 147 hospitals. The certified hospitals must meet various criteria for certification as an orthogeriatric unit (ATZ-DGU). These include interdisciplinary treatment by orthopedic surgeons and geriatricians, standard operating procedures for surgical treatment, pain management, mobilization, delirium assessment, osteoporosis assessment, and discharge management. In the registry, the initial data collection is based on standardized questionnaires from five different stages of treatment: at hospital admission, preoperative, intraoperative, after the first postoperative week, at hospital discharge, and optionally after 120 days. After 7 and 120 postoperative days, quality of life (QoL) is assessed using the validated EQ-5D-5L questionnaire [16]. The descriptive system comprises five dimensions: mobility, self-care, usual activities, pain/discomfort, and anxiety/depression. Each dimension has 5 levels: no problems, slight problems, moderate problems, severe problems, and extreme problems. The patient is asked to indicate his/her health state by ticking the box next to the most appropriate statement in each of the five dimensions. This decision results in a 1-digit number that expresses the level selected for that dimension. The digits for the five dimensions can be combined into a 5-digit number that describes the patient’s health state.

The standardized questionnaires of the ATR-DGU were developed together with the Fragility Fracture Network (FFN) and influenced by experiences from the “National Hip Fracture Database” of England and Wales and the “Australian and New Zealand Hip Fracture Registry” to provide internationally comparable data. The present study is in accordance with the publication guidelines of the ATR-DGU and registered as ATR-DGU project ID 2021-005. Approval for scientific data analysis from the ATR-DGU is granted via a peer-review process in accordance with the publication guidelines laid down by the AK ATR.

### 2.1. Patients

From 2016 to 2022, data from 60,254 patients were entered into the ATR-DGU. Because of the comparable execution of the early multiprofessional rehabilitation program in the federal states of Bavaria and Baden Württemberg, only patients from these regions were analyzed in this study. Furthermore, patients with periprosthetic or peri-implant fractures and additional injuries in need of treatment were excluded. Also, data sets with incomplete documentation were excluded. This reduced the number of potentially available patients that were treated in a certified orthogeriatric unit (ATZ-DGU) to 9580. These patients were divided into two groups, 4669 patients who received early multiprofessional rehabilitation (EMR) and 4911 patients with standard orthogeriatric co-management (OGC). 

EMR: Upon admission, patients with proximal femur fractures were treated according to the standard operating procedures for surgical treatment, pain management, early mobilization, delirium assessment, osteoporosis assessment, and discharge management. In addition, the patients received a standard geriatric assessment that included a medical assessment (cognition, emotion, self-help, mobility) and a social assessment (social environment, living environment, domestic activities, ambulatory care). Based on these initial geriatric assessments, an individual early multiprofessional rehabilitation treatment plan lasting at least 14 days was formulated. It consisted of a minimum of 20 therapeutic units (each 30 min) during the 2-week program. Patients were treated by a multiprofessional team consisting of physiotherapists, occupational therapists, specifically trained nurses, speech therapists, psychologists, and social workers. Nurses involved in the treatment of patients needed to be specially certified and trained with a focus on special geriatric patient care. The patients received daily geriatric visits. All members of the interdisciplinary team met on a weekly basis to discuss the status of the patient and to discuss further interventions.

OGC: Patients with standard orthogeriatric co-management were treated according to the standard operating procedures for surgical treatment, pain management, early mobilization, delirium assessment, osteoporosis assessment, and discharge management. The patients were visited at least twice a week by an interdisciplinary team of a geriatrician and an orthopedic surgeon. One unit of physical therapy was administered daily except on weekends.

### 2.2. Patient Matching

To control for differences between the demographics of the two groups (EMR vs. OGC), nearest neighbor matching was performed. Sex, ASA Physical Status Classification System (ASA) Score [17], Identification of Seniors at Risk (ISAR) score [18], fracture type, age, and walking ability before the fracture were considered as matching variables.

### 2.3. Exposure

The outcome was measured by the following parameters: walking ability 7 days and 120 days following surgery, reoperation rate at the time of discharge and after 120 days, rate of new bedsores at the time of discharge, in-house and 120-day mortality, EQ-5D-5L on the seventh day after surgery and on the 120-day follow-up, and the place of residency at the time of discharge and 120 days after the surgery. Other variables analyzed were the type of surgical procedures and anesthesia, full or partial weight bearing postoperatively, and length of stay.

### 2.4. Statistics

The statistical evaluation was performed with the statistical software R version 4.0.2. A 1:1 nearest neighbor matching using the Mahalanobis distance within a propensity score caliper (caliper width of 0.08) was conducted. Matching was performed using the MatchIt package (Ho, Imai, King, and Stuart, 2011) in R v. 4.3.2 (Foundation for Statistical Computing, Vienna, Austria). The covariates used in the matching were age, sex, ASA score, ISAR score, type of fracture, and walking ability before fracture. After matching, the absolute standardized mean differences of all covariates were less than 0.065, indicating that good balance was achieved. For descriptive analyses, categorical data are presented as counts and percentages and continuous variables as median with interquartile range (IQR). The results of the EQ-5D-5L questionnaire were transformed to a single index value using the German (GER) Ludwig value set Version 2.1 [19]. For the EQ-5D-3L questionnaire results, crosswalk mapping was performed using the German (GER) Ludwig value set to obtain EQ-5D-5L index values. These index values range from −0.661 for the worst to 1 for the best health status. Comparisons between the two groups (EMR vs. OGC) were made using the Χ^2^-test and Fisher’s exact test for categorical data and the Wilcoxon test for continuous data. Differences were considered statistically significant when *p* < 0.05.

### 2.5. Ethics

All patients consented in writing to the study. Written consent from all patients is a prerequisite to participate in the ATR-DGU. Without this, an entry into the register is not possible. The data from the ATR-DGU have received full approval from the ethics committee of the Philipps-University of Marburg (approval date: 5 April 2018, AZ/46/12). 

## 3. Results

A total of 9580 patients were available for evaluation for this study, 4669 patients who received EMR and 4911 patients with OGC. After the nearest neighbor matching method, finally, 8468 patients were compared with each other (Table 1). For the 120-day follow-up, the cohort was reduced to 5377 patients. 

Patients in the early multiprofessional rehabilitation group and patients with standard orthogeriatric co-management showed identical walking ability before the fracture and minimal differences in age, sex, fracture type, ASA, and ISAR score. In the EMR group, fewer patients lived in a nursing home preoperatively (EMR group 16.85% vs. OGC group 25.85%). Further pre-surgery data are shown in Table 2. 

Most procedures in each group were performed under general anesthesia. The two most common operational procedures were stabilization with a trochanteric nail and the implantation of a bipolar hip prosthesis. Time to surgery did not differ between the two groups. In the EMR group, slightly more patients were allowed to full weight bear postoperatively (*p* = 0.025) (Table 3).

### 3.1. Mortality

The mortality during the inpatient stay was significantly lower in the EMR group compared to the patients with OGC, 2.89% vs. 5.11% (*p* < 0.001). However, the mortality rate over the entire observation period up to 120 days showed no significant difference in both groups, 10.48% vs. 11.41% (*p* = 0.379). 

### 3.2. Walking Ability 

Significantly more patients who received early multiprofessional rehabilitation were able to walk independently or with help 7 days after surgery compared to patients in the standard orthogeriatric co-management group (86.44% vs. 77.78%, *p* < 0.001). The results showed that 120 days postoperatively, the walking ability leveled out. In the EMR group, 87.30% of the patients were able to walk either independently or with help compared to 85.84% in the OGC group. 

### 3.3. Complications and Reoperations

The reoperation rate during the inpatient stay was significantly higher in the EMR group compared to the OGC group, 3.64% vs. 2.51 (*p* < 0.001). In addition, the incidence of pressure ulcers was significantly higher in patients who received early multiprofessional rehabilitation, 3.64% vs. 2.46% (*p* < 0.001). 

### 3.4. Length of Stay, Discharge, and Living Situation Postoperative

The length of stay of patients in the multidisciplinary rehab program was significantly longer compared to the OGC group, 17.89 days vs. 12.44 days (*p* < 0.001). Hip fracture patients who received the early multiprofessional rehabilitation program were significantly less likely to be discharged to a nursing facility than patients in the OGC group, 19.7% vs. 25.87% (*p* < 0.001). A discharge to a geriatric rehabilitation clinic was significantly more frequent in the EMR group compared to the OGC group, 47.63% vs. 34.70 (*p* < 0.001). Three months after the hip surgery, we found no significant difference between the two cohorts concerning the number of patients who lived in a nursing home, 26.22% EMR group vs. 28.20% OGC group. In a sub-analysis of patients comparing patients living at home before the fracture and who did not die during their hospital stay, we found no differences concerning a discharge to a nursing facility, 9.82% EMR group vs. 9.29% OGC group. In the 120-day follow-up, we found that significantly more patients in the EMR group who previously lived in their own homes were placed in a nursing home compared to patients in the OGC group, 16.53% vs. 11.28% (*p* < 0.001).

### 3.5. Patient-Reported Outcome EQ-5D-5L

The patient-reported outcome measured by EQ-5D-5L showed, on average, significantly better results 7 days postoperatively for patients in the EMR group compared to the OGC group, 0.55 vs. 0.52 (*p* < 0.001). Additionally, 120 days postoperative, the patient-reported quality of life scores were, on average, balanced in both groups, 0.67 EMR group vs. 0.67 OGC. A full overview of the discharge, living situation, and the 7- and 120-day follow-up is displayed in Table 4 and Table 5. 

## 4. Discussion

Major chronic diseases are more prevalent in hip fracture patients compared to a sex- and age-matched population and the holistic treatment of these patients often exceeds the individual competence of an orthopedic surgeon [20]. The concept of a joint coordinated treatment by geriatricians and orthopedic surgeons was devised by Devas and Irvine in the late 1950s [21]. Several clinical trials and meta-analyses have investigated the effects of multidisciplinary orthogeriatric management on outcomes (e.g., mortality, physical function, institutionalization). In a landmark study, Prestmo et al. demonstrated improved functional outcomes and a lower rate of institutionalization in patients receiving comprehensive geriatric care following a hip fracture [14]. Their prospective randomized trial confirmed prior studies with fewer patients [22,23]. In addition to functional outcomes, orthogeriatric care is associated with a significant survival benefit. This was demonstrated by Rapp et al. in a large observational study with data from 58,001 patients [11]. What is more, orthogeriatric care has been shown to reduce readmission rates and prevent secondary fractures by enrolling patients in a fracture liaison service [11,12,13,24]. Although orthogeriatric care models vary from one hospital to the other, there are three main models designed to optimize the management of elderly hip fracture patients: treatment on an orthopedic ward with a geriatric consult, treatment on a geriatric ward with an orthopedic consult, or treatment on an integrated ward with joint orthopedic and geriatric responsibility for the patient. Several studies have investigated the effect of these different models on the outcomes following hip fracture [12,25]. In a systematic search comparing the different models, Kammerlander et al. demonstrated the lowest in-hospital mortality rate, lowest length of stay, and the lowest time to surgery in models with integrated care [25].

In addition, Gleich et al. were able to show that increased geriatric care, defined as more than two weekly visits compared to twice weekly geriatric consultation, had a significant impact on outcomes, with better 7-day walking ability and a higher initiation rate of osteoporosis treatment during the in-hospital stay. However, they were not able to prove an effect of increased geriatric care on mortality [26].

There are numerous studies that have investigated the impact of orthogeriatric care or different rehabilitation protocols on the outcomes following hip fracture surgery. However, the specific effect of multidisciplinary rehabilitation compared to orthogeriatric treatment has not been investigated so far in a large patient population. Therefore, this study sought to investigate the additional effect of geriatrician-led multidisciplinary rehabilitation on outcome parameters compared to standard orthogeriatric care.

This study revealed a significant reduction in in-house mortality when patients were treated by an early multiprofessional rehabilitation program compared to standard orthogeriatric co-management. The overall low inpatient mortality rate in both groups of this study, with 2.86% in the EMR groups and 5.11% in the OGC group, compares favorably to the literature [11,27]. In a well-suited study on almost 60,000 patients, Rapp et al. observed 30-day mortality following hip fracture of 10.32% in patients receiving orthogeriatric care and 13.36% in patients receiving standard orthopedic care [11]. The overall mortality seems to be decreasing in the last decades. Moyet et al., in a meta-analysis of publications from 1988 to 2015, reported an overall mortality of 17.7% [12]. The further reduction of mortality in our study could be due to the following factors: immediate attention and management of acute medical problems, prompt operation after hospital admission [28], the treatment of geriatric medical problems by geriatricians, and the multidisciplinary rehabilitation protocol. In addition, the patients in our EMR group received daily physiotherapy. We believe that the immediate mobilization played a significant part in reducing mortality following hip fracture suggesting a dose-dependent effect of rehabilitation effort. In our study, the reduced mortality in patients receiving EMR compared to OGC was limited to the early phase following surgery. This suggests that the difference in mortality is caused by interventions during the initial hospital stay confirming our earlier assumptions. 

The reduction in mortality is at the expense of a higher complication rate. Compared to the OGC group, significantly more patients in the EMR group had to be surgically revised because of surgical complications (2.51% OGC group vs. 3.99% EMR group) and in more patients, new occurring bedsores were detected (2.46% OGC group vs. 3.64% EMR group). We believe that the higher complication rate is explained by a more intense management of the patients with a high number of professionals involved in the treatment resulting in a high number of contacts with the individual patient during the hospital stay. It is possible that the “high concentration” of patient contacts resulted in the immediate detection of complications and the immediate notification of the surgeon in the case of developing complications resulting in revision surgery, thereby preventing possible lethal progress. In addition to earlier mobilization (see above), this would also explain the lower mortality in the EMR group. We presume that the slightly higher rate of pressure ulcers detected in the EMR group could also be attributed to this phenomenon because patients in the EMR group received a higher number of physiotherapy units—an intervention that is known to prevent pressure sores. The above higher complication rate/lower mortality paradox should be used to educate medical personnel monitoring patients with proximal femur fractures.

Early mobility is known to reduce the overall mortality and the incidence of in-hospital complications like pneumonia and delirium [29]. In addition, early physiotherapy has been shown to improve the patient’s short-term functional outcome [30]. In elderly patients, even short-term disuse can lead to sarcopenia, a permanent reduction of mechanical muscle function, and increased frailty [31,32,33]. Furthermore, even small improvements in mobility in frail patients with limited resources may be significant. Minimization of immobilization is therefore of paramount importance following hip fracture surgery. Patients in the EMR group received significantly more physiotherapy and occupational therapy on weekdays and mobilization on weekends by specifically trained nurses compared to patients in the OGC group who only received one unit of physiotherapy on weekdays. In addition, more patients were allowed to fully weight bear immediately following surgery in the EMR group. Interestingly, the functional benefit of EMR could not be fully maintained at the 120-day follow-up. We pose two hypotheses for this phenomenon. Patients suffering from fractures of the proximal femur are a relatively heterogenous group composed of a subgroup of frailer patients at risk for institutionalization and another subgroup of slightly fitter patients. It is possible that the currently used assessments are not able to adequately discriminate these two patient groups from one another, whereas clinical judgment is able to do so. It is conceivable that the size of both subgroups was balanced in the initial matching process and at the 7-day follow-up, whereas at the 120-day follow-up, more patients in the frailer subgroup were lost to follow-up, mitigating the difference between the two groups.

Another explanation could be that the superior functional status at day 7 was caused by specific early interventions that were not present at later stages, notably physical therapy during the hospital stay with a convergence of the functional status in the OGC and EMR groups at later stages. The phenomenon of a convergence of functional parameters can also be seen following operative treatment of vertebral fractures with ballon kyphoplasty and open reduction and internal fixation of distal radius fractures with superior functional results in the early phase following surgery and comparable functional results with conservative therapy in later stages [34,35]. 

There is increasing evidence that length of stay is not a sufficiently good parameter to assess the quality of treatment in hip fracture patients [33]. Nordström et al. observed that a shorter length of in-hospital stay (10 days or less) was associated with an increased risk of readmission and death after discharge and therefore could be detrimental to patient health [36]. However, analysis of data from the Swedish register showed that patients with a shorter stay did not show worse outcomes when they were discharged directly to their own homes and that the relationship between the length of stay and negative outcomes depends on the type of care received during the hospital stay and on the discharge destination [37]. In our study, patients in the EMR group were discharged, on median, significantly later than patients in the OGC group (17.1 days EMR group vs. 11.1 days OGC group), since the early multiprofessional rehabilitation program lasted for at least 14 days. The institutionalization rate of 16.53% in the EMR group and 11.28% in the OGC group was comparable to that in the literature. Rapp et al. documented an institutionalization rate of 15% for women and 12% for men [11] and Schoenberg et al. reported 16% for hip fracture patients who previously lived in their own homes [13]. One explanation for this unexpected result could be an indiscernible selection bias, with a preferred selection of frailer patients previously living at home for the early multiprofessional rehabilitation, although the groups were matched according to their pre-fracture ambulatory status. This hypothesis is supported by the findings of Neuerburg et al. and a metanalysis that also reported a higher percentage of institutionalization in patients receiving orthogeriatric care compared to standard orthopedic treatment [15,27]. 

The potential savings for insurances and the individual patients that were generated by the functional benefits of EMR must be weighed against the increase in hospital resource utilization. Because of the register-based study protocol with a large data set of anonymized data, a cost-effectiveness analysis was not possible. Further studies must elucidate the possible cost-effectiveness of MDR compared to OGC. In addition to analyzing the immediate costs and trade-offs of early interventions, further studies must also include the long-term resource utilization of ambulatory care and medical procedures provided outside the hospital in both groups.

Quality of life (QoL) was measured with the EQ-5D-5L and showed, on median, significantly better results for the EMR group 7 days after surgery (0.62 EMR group vs. 0.57 OGC group). This could be explained by the better early walking ability in the EMR group and the psychological effect of a more intensive treatment counteracting the feelings of helplessness and despair. Again, 120 days after the surgery, we found comparable median results in both groups (0.687 EMR group vs. 0.733 OGC group) with overall lower values compared to age-appropriate values for the general population [38]. Our 120-day postoperative EQ-5D-5L results are consistent with the literature. A multicenter prospective study from Spain reported an EQ-5D-3L score of 0.6 (IQR 0.1–0.8) 4 months postoperatively [39], confirming data from an international prospective study that reported a median of 0.54 (95% confidence interval (CI) 0.51–0.58) 4 months postoperatively.

The strength of this study is the large dataset of over 8000 patients and the exact documentation of the analyzed outcome parameters. Nevertheless, a registry analysis has some limitations. The number of included patients for each specific research question varies since only complete records were used and the 120-day follow-up is voluntary, explaining a follow-up rate of 48% in our study. Furthermore, it is quite possible that more cognitively and/or physically highly impaired people missed the 120-day follow-up. Therefore, a selection bias could have been introduced. Our matching criteria (sex, ASA score, ISAR score, fracture type, age, and pre-fracture walking ability) and the standardized data acquisition do not provide a fully comprehensive description of the patient’s health. The registry only provides information about surgical complications and the occurrence of pressure ulcers. Internal and neurological complications are not included. Knowing that medical problems in this group are often of a geriatric and not of a surgical nature, we were not able to examine if early multiprofessional rehabilitation is able to reduce these complications more effectively than standard orthogeriatric co-management.

## 5. Conclusions

This matched pair analysis of registry data demonstrates a lower in-house mortality and superior early walking ability in patients treated with a geriatrician-led multidisciplinary rehabilitation protocol compared to “standard” orthogeriatric management. These results support the concept of interdisciplinary rehabilitation. 

## Figures and Tables

**Table 1:** Patient cohort before and after matching.

Patient Cohort	*n*
Complete cases (before matching)	9580
Early multiprofessional rehabilitation	4669
Standard orthogeriatric co-management	4911
Cases after nearest neighbor matching	8468
Early multiprofessional rehabilitation	4234
Standard orthogeriatric co-management	4234

**Table 2:** Preoperative patient data, * *p* < 0.05.

	EMR *n* = 4234	OGC *n* = 4234
**Age at admission**		
Min/max	70/110	70/107
mean	84.65	84.34
**Sex % (*n*)**		
male	28.32 (1199)	27.47 (1163)
female	71.68 (3035)	72.53 (3071)
**ASA-Score & (*n*)**		
Median (IQR)	3 (3-3)	3 (3-3)
ASA 1	0.92 (39)	0.80 (34)
ASA 2	22.70 (961)	22.51 (953)
ASA 3	69.49 (2942)	69.84 (2957)
ASA 4	6.90 (292)	6.80 (288)
ASA 5	0.00 (0)	0.05 (2)
ISAR-Score %(*n*) *		
Median (IQR)	3 (2–4)	2 (1–4)
0	10.75 (455)	16.51 (699)
1	13.84 (586)	11.55 (489)
2	24.68 (1045)	22.51 (953)
3	24.02 (1017)	22.44 (950)
4	18.52 (784)	18.26 (773)
5	6.92 (293)	6.66 (282)
6	1.28 (54)	2.08 (88)
**Walking ability before fracture % (*n*)**		
Able to walk indepentdently or with help	98.11 (4154)	98.11 (4154)
Not able to walk	1.89 (80)	1.89 (80)
Living situation at time of fracture % (*n*) *		
Nursing residence	16.85 (706)	25.85 (1082)
Hospital	1.24 (52)	0.53 (22)
At Home	81.31 (3407)	73.05 (3058)
others	0.60 (25)	0.57 (24)
**Fracture type %(*n*)**		
medial/intracapsular	43.84 (1856)	45.65 (1933)
intertrochanteric	52.01 (2202)	50.64 (2144)
subtrochanteric	4.16 (176)	3.71 (157)

**Table 3:** Perioperative data, * *p* < 0.05, *** multiple answers possible.

	EMR	OGC
Surgical technique % (*n*) ***		
Cannulated srews	1.2 (50)	0.9 (38)
Dynamic Hip Srew	2.2 (93)	1.7(71)
Proximal Femur Nail	54.1 (2290)	53.5 (2266)
Bipolar Hemiprothesis	33.8 (1430)	35.8 (1517)
Total Hip Replacement	7.7 (324)	7.4 (314)
Anesthesia procedures % (*n*) ***		
General anesthesia	95.4 (4041)	90.2 (3817)
Spinal anesthesia	4.4 (186)	9.8 (417)
Others	1.4 (58)	2.5 (106)
**Time to surgery (in hours)**		
Median (IQR)	16.5 (7.7–23.1))	16.3 (7.6–23)
Full weight bearing postoperatively % (*n*) *		
Yes	94.70 (4004)	93.53 (3946)
No	5.30 (224)	6.47 (273)

**Table 4:** Discharge and postop. living situation, * *p* < 0.05.

	EMR	OGC
Length of hospital stay, surviving patients (in days) *		
Number of patients	4085	3996
Median (IQR)	17.1 (15–20.1)	11.1 (9.1–14.1)
Discharged from hospital to %(*n*) *		
Rehabilitation clinic	4.02 (170)	7.15 (302)
Geriatric rehabilitation clinic	47.63 (2012)	34.72 (1467)
Acute Geriatric hospital	8.12 (343)	7.41 (313)
Other Hospital	1.61 (68)	2.56 (108)
Nursing home	19.70 (832)	25.87 (1093)
Home/Assisted living	16.03 (677)	17.18 (726)
Death	2.89 (122)	5.11 (216)
Discharged to, survivors who previously live at home % (*n*) *		
Rehabilitation clinic	4.75 (158)	10.12 (295)
Acute geriatric hospital	8.74 (290)	7.82 (228)
Other hospital	1.75 (58)	2.71 (79)
Geriatric rehabilitation clinic	55.68 (1848)	46.54 (1357)
Nursing home	9.82 (326)	9.29 (271)
Home /assisted living	19.25 (639)	23.53 (686)
Living situation of survivors after 120 days % (*n*) *		
Rehabilitation clinic	0,00 (0)	0.06 (1)
Acute geriatric hospital	0.00 (0)	0.06 (1)
Other hospital	1.46 (27)	0.37 (6)
Geriatric rehabilitation clinic	0.76 (14)	0.31 (5)
Nursing home	29.29 (543)	31.83 (514)
Home	67.53 (1252)	66.13 (1068)
Others	0.98 (18)	1.23 (20)
Living situation of survivors who previously lived at home after 120 days % (*n*) *		
Rehabilitation clinic	0.00 (0)	0,22 (4)
Acute geriatrie/other hospital	1.47 (27)	0.49 (9)
Geriatric rehabilitation clinic	0.87 (16)	0.22 (4)
Nursing home	16.53 (303)	11.28 (206)
Home	80.14 (1469)	86.59 (1582)
Others	0.98 (18)	1.21 (22)

**Table 5:** 7- and 120-day follow-up data, * *p* < 0.05.

	EMR	OGC
Walking Ability (7 days post op) % (*n*) *		
Able to walk independently or with help	86.44 (3607)	77.78 (3185)
Not able to walk	13.56 (566)	22.22 (587)
**Walking Ability (120 days post op) % (*n*)**		
Able to walk indepentdently or with help Not able to walk	87.30 (1663) 12.70 (242)	85.84 (1425) 14.16 (235)
Died during inpatient stay % (*n*) *		
Yes	2.89 (73)	5.11 (216)
No	97.11 (4102)	94.89 (4009)
**Died during follow up of 120 days % (*n*)**		
Yes	10.48 (217)	11.41 (208)
No	89.52 (1854)	88.59 (1615)
Reoperation during inpatient stay % (*n*) *		
Yes	3.99 (169)	2.51 (106)
No	96.01 (4063)	97.49 (4122)
**Reoperation during follow up of 120 days % (*n*)**		
Yes	3.62 (72)	3.08 (53)
No	96.38 (1942)	96.92 (1667)
Pressure ulcers during inpatient stay % (*n*) *		
Yes	3.64 (154)	2.46 (104)
No	96.36 (4080)	97.54 (4130)
EQ-5D-5L (7 days post op) *		
Number of patients (*n*)	3455	3355
Median (IQR)	0.62 (0.402–0.687)	0.572 (0.336–0.687)
**EQ-5D-5L (120 days post op)**		
Number of patients (*n*)	1719	1559
Median (IQR)	0.687 (0.515–0.859)	0.733 (0.505–0.859)

## Data Availability

Restrictions apply to the availability of these data. Data were obtained from the Registry for Geriatric Trauma (ATR-DGU) and are available from the Academy for Trauma Surgery (AUC) with the permission of the Working Committee on Geriatric Trauma Registry (AK ATR) of the German Trauma Society (DGU).
